# Paper-Based Immunosensors with Bio-Chemiluminescence Detection

**DOI:** 10.3390/s21134309

**Published:** 2021-06-24

**Authors:** Maria Maddalena Calabretta, Martina Zangheri, Donato Calabria, Antonia Lopreside, Laura Montali, Elisa Marchegiani, Ilaria Trozzi, Massimo Guardigli, Mara Mirasoli, Elisa Michelini

**Affiliations:** 1 Department of Chemistry “Giacomo Ciamician”, University of Bologna, 40126 Bologna, Italy; maria.calabretta2@unibo.it (M.M.C.); martina.zangheri2@unibo.it (M.Z.); donato.calabria2@unibo.it (D.C.); antonia.lopreside2@unibo.it (A.L.); laura.montali2@unibo.it (L.M.); elisa.marchegiani2@unibo.it (E.M.); ilaria.trozzi2@unibo.it (I.T.); massimo.guardigli@unibo.it (M.G.); 2 Center for Applied Biomedical Research (CRBA), University of Bologna, 40138 Bologna, Italy; 3 Interdepartmental Centre for Renewable Sources, Environment, Sea and Energy (CIRI FRAME), Alma Mater Studiorum, University of Bologna, 48123 Ravenna, Italy; 4 INBB, Istituto Nazionale di Biostrutture e Biosistemi, Via Medaglie d’Oro, 00136 Rome, Italy; 5 Health Sciences and Technologies-Interdepartmental Center for Industrial Research (HST-ICIR), University of Bologna, 40126 Bologna, Italy

**Keywords:** paper-based, bio-chemiluminescence, immunosensor, biosensor, point-of-care

## Abstract

Since the introduction of paper-based analytical devices as potential diagnostic platforms a few decades ago, huge efforts have been made in this field to develop systems suitable for meeting the requirements for the point-of-care (POC) approach. Considerable progress has been achieved in the adaptation of existing analysis methods to a paper-based format, especially considering the chemiluminescent (CL)-immunoassays-based techniques. The implementation of biospecific assays with CL detection and paper-based technology represents an ideal solution for the development of portable analytical devices for on-site applications, since the peculiarities of these features create a unique combination for fitting the POC purposes. Despite this, the scientific production is not paralleled by the diffusion of such devices into everyday life. This review aims to highlight the open issues that are responsible for this discrepancy and to find the aspects that require a focused and targeted research to make these methods really applicable in routine analysis.

## 1. Introduction

Immunoassays have been routinely used in laboratories equipped with bulky instrumentation and skilled personnel for the quantification of target analytes for various applications in healthcare, food safety and environmental monitoring. In recent years, research has mainly focused on making the immunoassay technique suitable for portable analytical formats, to perform low-cost tests directly on site [1]. Indeed, thanks to their selectivity and specificity, immunoassays allow the detection of analytes within complex matrices without the need for long-processing sample pretreatments. Among portable formats suitable for the development of point-of-care (POC) immunoassays, the paper-based approach has attracted strong interest thanks to its advantageous features, including low cost, straightforward procedures, flexibility of the paper-based support, short turnaround time and small consumption of samples and reagents. For these reasons, paper-based POC immunoassays are expected to become a feasible option for monitoring human healthcare, food safety or water quality in resource-limited settings and, thanks to the recent improvements in microfluidics and nanotechnologies, they have developed rapidly in recent years [1]. Among the different varieties of paper-based format biosensors, those combined most frequently with immunoassays are lateral-flow immunoassays (LFIA) and microfluidic paper-based devices (µPADs) [2].

LFIA is an integrated platform in which pre-stored reagents are entrapped on a nitrocellulose strip. By adding the sample, these reagents are solubilized and, while they are flowing through the membrane, bioassays take place. On the surface of the nitrocellulose strip, different areas contain specific probes responsible for the recognition and detection of the target analyte. It is possible to exploit different bioassay formats, including sandwich, competitive or multiplex formats.

As an alternative, μPADs are characterized by the presence of hydrophilic and hydrophobic microchannels, which allow to develop different designs suitable for the desired application. The microfluidic pattern is generally obtained by exploiting chemical printing and/or cutting and it is possible to develop 2D or 3D configurations for moving fluids through vertical and horizontal pathways [2,3].

The analytical performance of miniaturized analytical devices represents the bottleneck when high sensitivity is required, such as for the detection of analytes present at trace levels in complex matrices [4,5]. For this reason, one of the most important aspects in the development of these methods is the selection of the detection technique since it has to combine high sensitivity with reduced sensing equipment, providing the possibility of working with portable and low-cost devices and ease of operation even by non-specialized personnel [5]. Several attempts to avail popular detection methods. such as UV/Vis absorption spectroscopy [6,7,8,9], have been reported in literature but the most promising techniques are those based on electrochemical and luminescence detection.

In particular, chemical luminescence is based on the production of photons triggered by a chemical reaction, as for chemiluminescence (CL), and, when the reaction occurs within living organisms, the phenomenon is called bioluminescence (BL). Since BL and CL reactions start in the dark, photons can be measured with high efficiency ensuring the absence of nonspecific signals, thus avoiding the background commonly encountered with photoluminescence measurements. For this reason, CL-based analytical methods can achieve, in principle, high detectability and can represent an alternative or a complementary approach in the field of optical biosensors [10].

**Figure 1:**
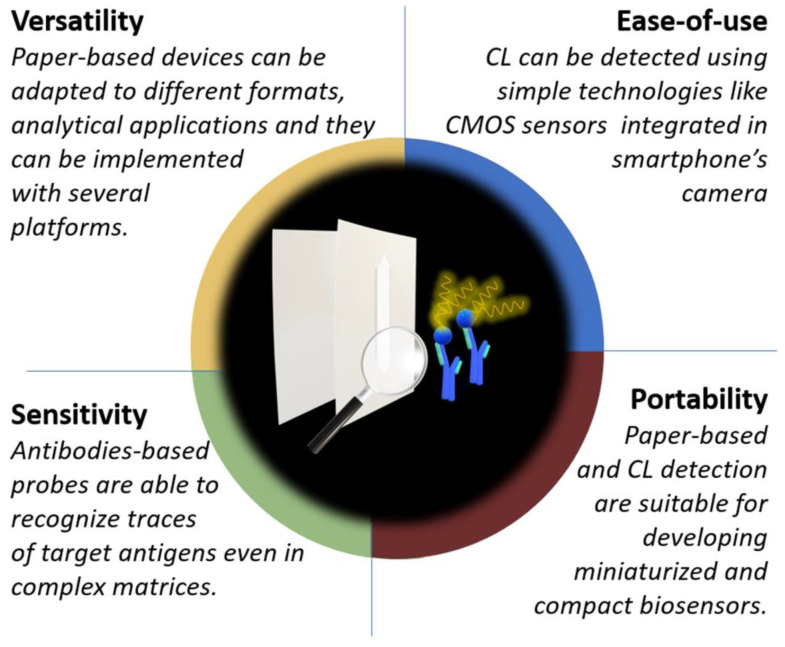
Schema of the advantages obtained by combining paper-based immunosensor with CL detection.

Together with their high sensitivity, CL and BL allow to work with a wide linear range, no radioactive reagents and simple equipment making them an ideal detection principle for POC analytical instrumentation [11]. Figure 1 schematizes the advantages in combining paper-based immunosensors with CL and BL detection.

**Scheme 1:**
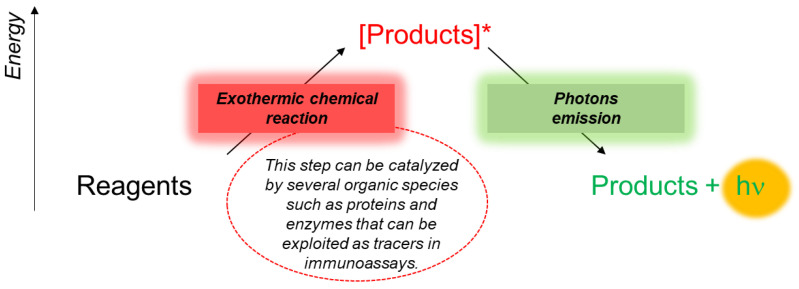
Schematic representation of bio-chemiluminescent reactions.

The photons produced by CL reactions are due to the returns of a molecule to the ground state after the excitation to the singlet excited state reached through an exothermic chemical reaction (Scheme 1). 

The wavelengths typical of the CL emission are in the range of the visible and infrared spectrum [12,13]. Since it occurs in living organisms, the BL phenomenon is generally based on the catalytic activity of enzymes (e.g., luciferases) or photoproteins [14]. BL and CL detection principles can be used in immunoassays by exploiting an enzymatic-label conjugate to an immunoreagent and a specific BL or CL substrate. Using either BL or CL, a significative amplification of the analytical signal (about 10^4^–10^5^ times) can be achieved thanks to the characteristic enzymatic turnover [15].

Horseradish peroxidase (HRP) is probably the most used CL label. HRP is able to catalyze the oxidation of luminol in the presence of a peroxide which leads to the formation of aminophtalate in a singlet excited state. The decay of this species to the ground state brings about the release of photons at a characteristic wavelength (428 nm) [16]. HRP shows good stability combined with a high enzymatic turnover number, making it an excellent candidate for CL-based biospecific assays. By adding to the CL cocktails suitable enhancers acting as electron transfer mediators (e.g., substituted phenols, substituted boronic acids, indophenols, N-alkyl phenothiazines [17] and 4-dialkylaminopyridine [18]), it is possible to increase the signal intensity and to stabilize the CL signal over time.

Firefly luciferase is the most exploited BL protein and it acts by catalyzing the formation of luciferyl-adenylate (LH_2_-AMP) from D-luciferin (D-LH_2_) and ATP. LH_2_-AMP is then involved in a multi-step oxidative process that leads to the formation of an excited product which drives the emission of photons at 550–570 nm. While firefly luciferase emission can be modulated by changing the environmental conditions (e.g., pH, temperature, light emissions of BL), reactions involving click beetles and rail-road worms do not depend on pH and temperature [19]. A significant advantage of using BL reactions is the high quantum yield of luciferase-catalyzed reactions (i.e., 44% for *Photinus pyralis* luciferase) that provide unbeatable sensitivities [20]. Exploiting genetic fusion of the luciferase to an antibody fragment and chemical conjugation of luciferases to monoclonal antibodies, several BL antibodies were used in bioanalytical assay and imaging techniques [21]. Add-and-read homogeneous immunoassays using the NanoLuc Binary Technology (NanoBiT), a protein complementation system based on NanoLuc luciferase, were successfully developed to monitor multiple signaling pathways’ activation [22] and to detect the mycotoxin fumonisin B1 [23].

Currently, the coupling of these methods with new nanomaterials (e.g., gold nanoparticles, quantum dots and magnetic materials) promoted a great breakthrough in the effectiveness of these techniques [24,25].

From the point of view of signal enhancement, several improvements made these techniques suitable for the ultrasensitive detection of target analytes, even in paper-based biosensor formats [4,26]. Despite this, however, there are still open issues that need to be further investigated to promote a wider use and diffusion of CL/BL paper-based immunoassays for POC applications.

One of the main issues is certainly due to the fact that such bioassays rely on bioreagents (e.g., enzymes and small-molecule substrates) that are not stable and require dedicated shipping and storage conditions such as dry ice and special packaging. These bioreagents can be affected by thermal denaturation and/or chemical modification, oxidation or hydrolysis processes [27,28]. The requirement of a strict cold chain hampers their use for on-site applications, especially in remote areas and developing countries [29]. For these applications, the implementation of BL is critical since luciferases and most of their substrates are very sensitive to environmental conditions and are often characterized by a short shelf-life, thus requiring very precise storage temperatures. For this reason, the use of BL-immunoassay in the field of paper-based biosensors is quite limited.

A promising approach has been recently reported by Hall et al. who developed a shelf-stable BL homogenous immunoassay reagent, in which all components were immobilized in a lyophilized cake, based on a Nanoluc complementation reporter system [30].

Another limitation to the use of CL and BL detections for paper immunoassays is connected to the requirement of substrate for triggering the luminescent reaction, making more complex the design of the analytical device and increasing its cost.

The purpose of this review is to introduce the newest and most significant progress in the field of biosensors based on immunoassays coupled with paper format and CL-BL detection principle, evaluating the great improvements in sensitivity thanks to the use of innovative materials and enhancers and critically highlighting the main hurdles to real-life applicability in order to promote a targeted research for making these devices ready for their effective use.

## 2. Nanomaterials for Signal Enhancement of Chemiluminescence

The main limitations of CL-based detection are linked to the weakness of the light signals and short luminescence time [31]. Conventionally, for increasing the intensity of the signals and the stability of light emission over time, enhancers have been used to increase the sensitivity for CL-based immunoassays [32]. The aim is to achieve similar analytical performance of standard laboratory-based immunoassays which are able to detect target analytes even at the apto- zepto-molar levels with wide dynamic ranges (up to six orders of magnitude) [33]. In addition to the continuous progress made regarding enhancers development, different nanomaterials have recently been proposed as catalysts or chemicals carriers for enhancing CL signals [34,35]. In Table 1 the main strategies for enhancing the CL signal exploiting nanomaterials are reported. Metal nanoparticles (MNPs)-based enhanced CL have been extensively studied thanks to the strong catalytic properties of MNPs, such as silver (Ag), gold (Au) and platinum (Pt), that allow to increase the surface area and surface electron density in CL reactions [36]. Furthermore, the MNPs are very frequently used for the development of paper-based devices as they are easy to immobilize by absorption and they are effectively re-solubilized with the flowing of a liquid phase. AuNPs have been widely employed since they combine the catalysis of the CL reaction and the easiness of conjugation with different kinds of chemicals and biomolecules (e.g., luminol, enzymes, antibodies, DNAzymes, etc.). Recently, Han et al. [37] developed a CL-LFIA biosensing platform incorporating AuNPs into a polymer-networked HRP with an antibody (Au-polyHRP-AB) as a new scheme for enhanced enzyme conjugation. This approach involved a mass-producible and time-programmable amplification process based on a water-swellable polymer that allows to automate sequential reactions (immunoassay and signal amplification). The authors developed a specific part of the analytical device (amplification part) using the water-swellable polymer as a fluid switch and integrated it into the platform for triggering automated sequential reactions. The amplification part was produced using fabrication methods (lamination, cutting and assembly) that are widespread and well known in the LFIA industry. The test strip and amplification part were then integrated into a single device through a comprehensive housing assembly. The developed platform was used to quantitatively evaluate cardiac troponin I (cTnI) in serum samples, within 20 min obtaining a detection range of six orders of magnitude and a detection limit of 0.84 pg mL^−1^ when compared to the standard laboratory equipment, making it suitable for clinical use.

Hua Cui’s group proposed the simultaneous determination of three acute myocardial infarction (AMI) biomarkers by a three-dimensional (3D) μPAD exploiting a time-resolved CL emission approach [38]. They immobilized on the test zone a primary antibody functionalized with AuNPs (Ab1-AuNPs), and a secondary antibody labeled with both Co(II) catalyst luminol and AuNPs (Co(II)-Ab2-luminol-AuNPs). The CL activity of Co(II)-Ab2-luminol-GNPs was due to the simultaneous catalytic effect of Co(II) and GNPs. Indeed, Co(II) catalyzed the decomposition of H_2_O_2_, generating the hydroxyl radical ·OH and a luminol radical which further reacted with O_2_ to produce an O_2_^−^ radical. The catalytic effect of Co(II) was enhanced by the coordination of Co(II) to the surface of luminol-GNPs. The reaction between the luminol radical and O_2_^−^ radical was responsible for the strong CL emission. Another effect promoting a further amplification was the presence of COO^-^ groups in Ab2 and BSA that could also react with the O_2_^−^ radical, forming reactive −CO_4_^2−^ radicals. Moreover, GNPs could also bind a large number of luminol molecules and Co(II) catalyst, resulting in further enhanced CL signal.

CL immunoreactions were performed at three detection zones by assembling Ab1-AuNPs, antigen and Co(II)-Ab2-luminol-AuNPs. Thanks to the flow of H_2_O_2_ to different detection zones at different times, CL signals were temporally resolved allowing a multiplexing analysis format.

**Figure 2:**
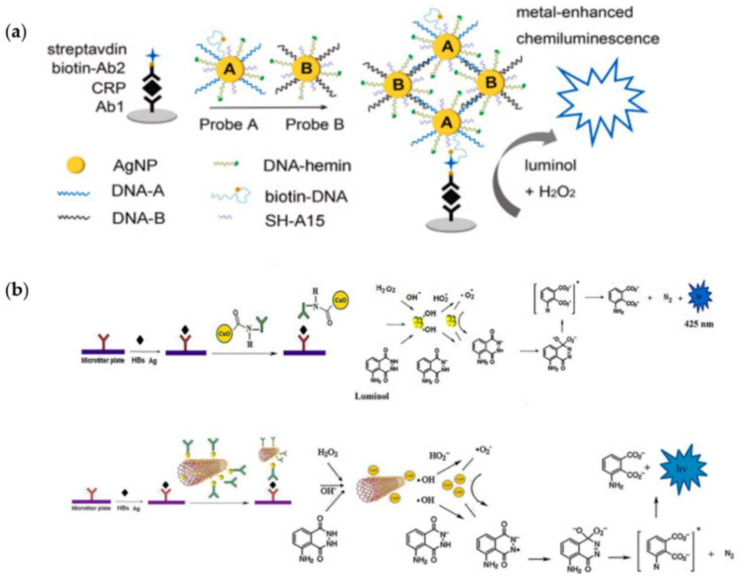
Nanomaterials for CL signal enhancement: (**a**) a novel silver nanoparticle hybrid probe designed for ultrasensitive metal-enhanced CL immunoassay of the marker of cerebrovascular disease. Reprinted with permission from ref. [39]. Copyright 2019 Elsevier.; (**b**) a sandwich-type CL immunoassay using the as-prepared CuONPs–Ab conjugate and a sandwich-type CL immunoassay using the as-prepared Ab-MWCNT/CuONPs conjugate. Reprinted with permission from ref. [49]. Copyright 2017 Elsevier.

Another example of MNPs-enhanced CL was reported by Zong et al. [39] who developed an immunoassay for the detection of C-reactive protein (CRP) exploiting two AgNP probes, i.e., probe A, composed of DNA-hemin/DNA-A/biotin-DNA modified with AgNPs, and probe B, consisting in DNA-hemin/DNA-B modified with AgNPs. By DNA-A and DNA-B hybridization, a CL signal was generated. Thanks to the high content of hemin molecules, the AgNP hybrid probes showed excellent CL signal amplification, allowing a detection limit for CRP down to 0.05 ng·mL^−1^ (Figure 2a).

In addition to MNPs, quantum dots (QDs), have gained great attraction as catalysts of CL reactions [40]. In particular, they act in two different steps: firstly, they take part in the decomposition of H_2_O_2_ to generate free radicals, and secondly, they promote CL by energy transfer and electron transfer annihilation effects. Since QDs enhance the CL signal by serving as the direct CL emitters/catalyzer of redox CL reactions, they are usually employed for the development of electrochemiluminescent (ECL)-based biosensors. In this case, the light is produced as a result of a chemical reaction triggered by either direct oxidation of CL reagents or indirect enhancing/inhibitory effects of certain luminescent compounds [41]. For this reason, several QD-ECL-based biosensors have been reported in literature for the detection of proteins [42,43], small molecules [44] and cells [45].

As an alternative to conventional QDs, carbon nanomaterials (CNMs) have emerged to improve CL systems, thanks to their low toxicity, environmental friendliness, low cost and simple synthetic routes [46]. In particular, carbon nanoparticles (CNPs), graphene, graphene oxide (GO) and carbon nanotubes (CNTs), possess unique optical, catalytic and biocompatible properties.

A paper-based CL immunodevice was described for sensitive determination of the carcinoembryonic antigen by Chen et al. [47]. Capture antibody was immobilized on the paper-based chip following a plasma treatment of the paper surface. A detection antibody was obtained by its labeling with carbon nanospheres functionalized with HRP (multi-HRP-HCS-Ab2). The authors exploited highly carbonized nanospheres (HCS), which showed abundant carboxyl groups on their surface, functioning as an ideal carrier for signal molecule loading. Multiple enzymes can be immobilized on HCS through carboxyl groups leading to signal enhancement. In this case, the detection antibody, multi-HRP-HCS-Ab2, was not pre-stored on the paper-surface but it was added by the operator after the sample incubation during the detection step.

TiO_2_ nanoparticles coated multiwalled carbon nanotubes (TiO_2_/MWCNTs) were synthesized as an amplification catalyst label by Li et al. [48]. A capture antibody was immobilized on modified-chitosan paper membrane and, after the recognition reaction, the TiO_2_/MWCNTs were used as catalysts for the luminol-*p*-iodophenol-H_2_O_2_ CL system, producing an enhanced CL emission. They applied the developed bioassay to detect prostate-specific antigen demonstrating a good linear response range from 0.001 to 20 ng/mL with a detection limit of 0.8 pg/mL.

Another study reported the comparison of CuO nanoparticles and CuO/MWCNT nanocomposites as enhancers for CL immunoassays for detection of the hepatitis B surface antigen [49]. CuO nanoparticles and CuO/MWCNT nanocomposites significantly enhanced the luminol CL intensity, and the detection limits (1.8 and 0.85 ng·mL^−1^, respectively) were comparable with those obtained with a clinical routine CL immunoassay (Figure 2b).

Currently, the combination of nanomaterials-based enhanced CL-immunoassays with paper platform is still at the proof-of-concept stage, but the interesting results reported in literature show that it is a promising strategy for developing a great variety of sensitive analytical methods and further in-depth studies will be required to identify the best approaches.

## 3. Fluid Control and Fluid Handling

**Figure 3:**
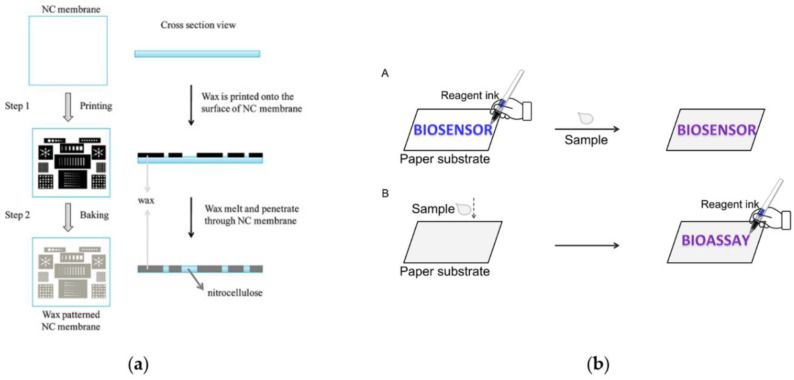
Fluid control and fluid handling: (**a**) schematic illustration of processes to fabricate paper-based microfluidics in an NC membrane by wax printing. It includes mainly two steps: step 1 is to print the wax pattern onto the surface of the NC membrane with a wax printer; step 2 is to bake the wax-printed NC membrane in an oven at 125 °C for 5 min to let the wax melt and penetrate through the membrane fully. Reprinted with permission from [55]. Copyright (2010) American Chemical Society. (**b**) Design of ”pen-writing” technique. (A) When writing biosensors on paper, the sensing elements are directly written to form patterned paper biosensors and would be expected to function at the initial location after introduction of samples leading to the fact that the colored product may not stick in the area where these biomolecules are written. (B) The proposed approach is not dependent on hydrophobic barriers to define the patterning of paper substrates, providing an alternative for sensor fabrication. Paper substrates are directly employed for the collection and transportation of samples, and then the reagent ink pen is used for sample analysis Reprinted with permission from [56]. Copyright 2019 Elsevier.

Microfluidic paper-based (μPADs) analytical devices provide an alternative platform for liquid transport via capillary forces without the need for external pumps [52] and they can be easily implemented for multiplexed analysis by simply adding channels [3]. Moreover, hydrophobic patterning can be employed to spatially confine the flow of hydrophilic solvent including biofluids [53]. Fabrication techniques include photolithography, wax printing, screen printing, inkjet printing and plasma oxidation [54]. The use of wax as the blocking material to construct microfluidic platforms (Figure 3a) has become a major trend; indeed it guarantees simplicity, rapidity, low-cost, suitability for producing prototypal µPADs on a large scale [55]. Among the different methods for wax patterning (including use of wax pens, screen printing and direct printing by wax printers), painting with a wax pen is the simplest method since hydrophobic barriers (Figure 3b) are easily obtained and characterized by high flexibility [56].

Yang et al. used a crayon and a pencil to construct a hand-drawn and written pen-on-paper electrochemiluminescence immunodevice for POCT [57]. Using wax printing technology, Wang et al., developed a portable analytical device based on CL immunoassay integrated into a low-cost μ-PAD by covalently immobilizing capture antibody on a chitosan membrane [58]. Exploiting tumor markers as target analytes and paper microzone plate as platform, the application of the proposed system was successfully optimized achieving a linear range of 0.1–35.0 ng mL^−1^ for α-fetoprotein, 0.5–80.0 U mL^−1^ for cancer antigen 125 and 0.1–70.0 ng mL^−1^ for carcinoembryonic antigen. The combination of chitosan modification and wax-screen-printing methodology for μPADs can be applied to other signal reporting approaches and other receptors for detecting different target analytes (such as DNA, proteins and small molecules) fitting the POCT purposes. Another covalent fabrication strategy focused on the activation of μPADs by periodate oxidation, which can form covalent bonds between polysaccharides and proteins, was exploited by the same group [59]. This strategy was used to covalently immobilize antibodies on paper while the wax-printing technology was employed for defining the reactive area of the μPAD.

Another protein immobilization method was proposed by Zhao et al. [60] based on plasma treatment of paper for the development of a low-cost immunosensor. The antibody was immobilized on the paper surface after 4 min oxygen plasma treatment. Plasma treatment allowed to produce an aldehyde group which was necessary for the direct immobilization of the antibody without any additional pretreatment. A sandwich CL immunoassay method was developed for carcinoembryonic antigen (CEA) detection in human serum obtaining a linear range of 0.1–80.0 ng/mL and a detection limit of 0.03 ng/mL.

Liu et al. proposed a CL immunoassay-based device developed by a craft-cutter to define flow channels, followed by lamination [61]. This approach for fabricating µPAD by cutting/lamination shares great similarities with the procedures employed for making an identification card. The procedure is very simple and offers a valid alternative to conventional methods employed for patterning paper using wax. They also proposed a protocol based on localized incision and paper-folding to separate the detection zone from flow channels. This trick eliminated possible reagent diffusion and flow during antibody immobilization steps and the requirement for washing steps. By incorporating luminol-based CL for detecting HRP-conjugated cotinine, they detected cotinine in mouse serum using competitive immunoassay. Thanks to the peculiarities of the proposed device, a 2D or 3D structure was obtained enabling both vertical and horizontal liquid flow. This feature allows tuning according to diagnostic requirements and simplifies multiplexed analyses [2].

Ge et al. [62] exploited the origami-based approach in order to combine on the same platform a system for blood plasma separation from whole blood, the automation of rinse steps and multiplexed CL detections. A 3D origami-based device was developed composed of a test pad surrounded by four folding tabs that could be patterned and fabricated by wax-printing on paper. In the proposed work, a sandwich-type CL immunoassay was developed, allowing to separate the operational procedures into several steps triggered by folding the pad and the addition of reagents/buffer. The developed 3D origami-based CL immunodevice was combined with a luminol-H_2_O_2_ CL system catalyzed by Ag nanoparticles and it was demonstrated that the excellent analytical performances allowed the simultaneous detection of four tumor markers. The same origami-based approach exploiting wax printing technology was recently proposed for the development of a multienzyme CL foldable paper-based biosensor for on-site detection of acetylcholinesterase (AChE) inhibitors [63].

To summarize, considerable progress has been achieved in the adaptation of existing batch and flow analysis methods to μPAD format in the field of CL-based assays. It is possible to imagine that this trend will continue unabated and soon a wide variety of μPADs will be developed for the detection of different biomarkers.

As it concerns fabrication methods, they should be as simple and cheap as possible and suitable for scaling up device production. In this context, the most employed methods for developing μPADs are based on wax printing and paper cutting which are low-cost and easily applicable on a large scale. The widespread adoption of these devices requires some advances in some crucial issues such as a proper validation and adequate long-term storage stability.

The use of mobile phones and tablets for signal detection and real-time processing of data could significantly strengthen the portability and user-friendliness of μPADs, making them accessible to both chemists and non-chemists.

## 4. Progress in Reagents Storage and Self-Contained Devices for POC Application

Conventional microfluidic devices rely on multiple-steps procedures for completing an analysis, posing a limit in their use for untrained personnel. The possibility to pre-store all the necessary reagents in “self-contained” POC devices is an emerging subject of study since it should bypass current limitations of BL and CL sensors [64]. The reagents related to POC testing comprise different chemical and biochemical species, including antibodies, enzymes, substrates and buffer solutions [65]. Different reagents require different storage and manipulation conditions. An ideal self-contained POC device can store all the reagents stably and release them easily and controllably. Paper can provide a low-cost platform for diagnostics, but the instability of biological molecules, such as proteins and enzymes immobilized on this support, can severely limit its commercial development. Indeed, the low stability of such biomolecules impedes the obtainment of biosensors that are stable during storage and shipping, as required by the market. CL-based immunoassays are based on the use of labile enzymes and substrates that need special care, challenging their routine employment for POC or field applications. For such assays, maintaining the long-term stability of enzymes and signal-generating small molecules remains a significant challenge, since reagents should work in extreme conditions far away from the well-controlled lab environments [66].

Antibodies play a crucial role in current bioactive paper-based diagnostics [58,67,68]. In particular they are widely employed in LFIA where they are immobilized in specific areas of the nitrocellulose membrane called test and control lines. The interaction between proteins and the nitrocellulose membrane is initially based on electrostatic interactions. Then, a combination of hydrophobic and hydrogen bonds allows the establishment of long-term bonding. There are several factors that can affect the binding process, such as reagent choices (the presence of non-specific proteins, materials that interfere with hydrogen bonds, materials that interfere with hydrophobic interactions, etc.), environment (e.g., humidity, temperature), processing methods (e.g., dispensing methods, drying methods) [69]. There is an extensive literature on methods for improving the stability of antibodies immobilized on different paper-based materials, including covalent binding [70], addition of preservatives for stabilizing the protein [71], plasma treatment of paper [60], leading to increased shelf life of the immunoreagents with unaltered immunological activity.

**Figure 4:**
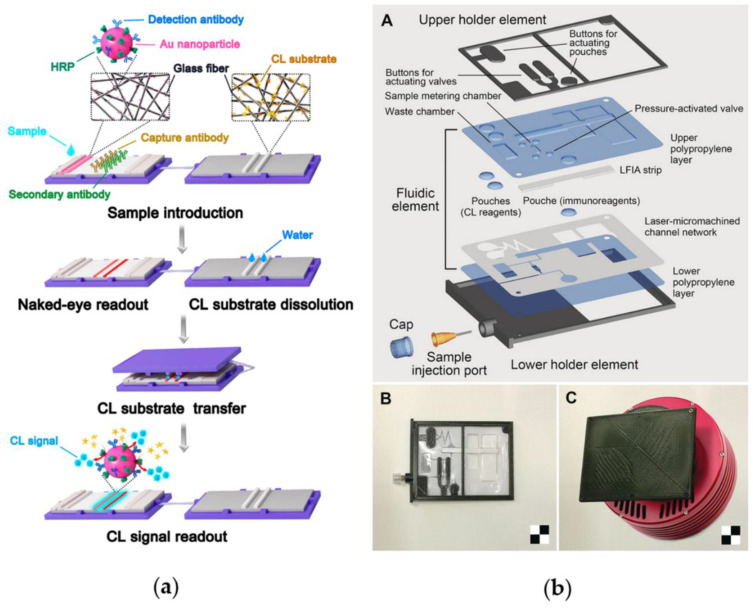
Self-contained devices for POC application: (**a**) a self-contained and easily processable CL lateral flow assay for POC testing. Reprinted with permission from ref. [64]. Copyright 2019 WILEY-VCH Verlag GmbH & Co. KGaA, Weinheim; (**b**) CL lateral flow immunoassay portable device to simultaneously detect two proteins (collagen and ovalbumin) for diagnostic campaigns on paintings: (A) Layout and (B) image of the disposable analytical cartridge; (C) CCD camera with 3D-printed mini dark box. Scale checkerboard is 2 × 2 cm. Reprinted with permission from ref. [76]. Copyright 2018 Wiley-VCH Verlag GmbH & Co. KGaA, Weinheim.

Despite recent advances in immobilizing proteinaceous biomolecules on paper through different strategies, the implementation of a CL-immunoassay, comprising antibodies, enzyme-labeled reagents and CL substrate into a unique paper platform, is still little explored. Deng et al. developed a self-contained and easily processable CL-LFIA comprised of three parts: the LFIA strip, the substrate pad and a polycarbonate (PC) holder [72]. In this case, the label employed for the immunoassay was composed of AuNPs immobilized on the conjugate pad labeled with antibody and HRP, simultaneously (Figure 4a). In this work, H_2_O_2_ was replaced by sodium perborate as oxidant of CL reaction and the CL substrate was lyophilized on the glass fiber for the assembly of the CL-LFIA. After performing the test, substrate was dissolved with deionized water and the substrate pad covered the LFIA for a short time to transfer the substrate to the NC membrane. The substrate mixture reacted under the catalysis of HRP and generated a CL signal for quantitative detection. In this case, the user must add to the biosensor only the sample and, during the detection step, water to dissolve the lyophilized CL substrate.

Alternatively, it is possible to build “self-contained” devices which integrate all the reagents necessary for performing the test in a microfluidic chip. On-chip reagents storage replaces manual reagents introduction, simplifying the detection process and reducing potential contamination risks.

Recently, we [73] reported self-contained CL-LFIA for salivary cortisol quantification composed of a disposable 3D-printed plastic cartridge (which contains a fluidic element with the LFIA strip, reagents reservoirs and valves manually activated), and a CL reader based on an ultrasensitive cooled CCD camera employed in a “contact imaging” configuration [74,75]. The analysis protocol was based on a simple manual procedure, started by operating valves and buttons to activate flow of sample and reagents which is then sustained by capillary forces. This biosensor was successfully used by the Italian astronaut Paolo Nespoli during the VITA mission (July–December 2017), thus demonstrating the possibility of performing sensitive CL-LFIA analyses directly onboard the International Space Station even in microgravity conditions [73]. CL-LFIA portable devices were also developed to simultaneously detect two proteins (collagen and ovalbumin) for diagnostic campaigns on paintings [76]. Since the immunological method required a multistep analytical protocol with sequential additions of sample and reagents, a disposable analytical cartridge was developed to streamline the procedure. In particular, the developed cartridge was ad hoc designed to contain the LFIA strip and all necessary reagents; thus, the analysis only required sample addition (Figure 4b).

## 5. Light Detection Technologies Integrated with CL Paper-Based Immunoassay Devices

Together with the enhanced CL system based on the use of innovative nanomaterials, the technologies related to the ultra-sensitive detection of photons have also improved considerably in the last decade. Indeed, the main requisite of CL measurements is the ability to collect as much light as possible to achieve the highest detectability and there are no limitations posed by specific optics geometry [14]. Several portable and easy-to-use detectors were proposed for the integration with paper-based compact analytical devices [77,78]. The main technologies for CL detectors implemented into portable immunosensors paper-based are reported in Table 2.

**Figure 5:**
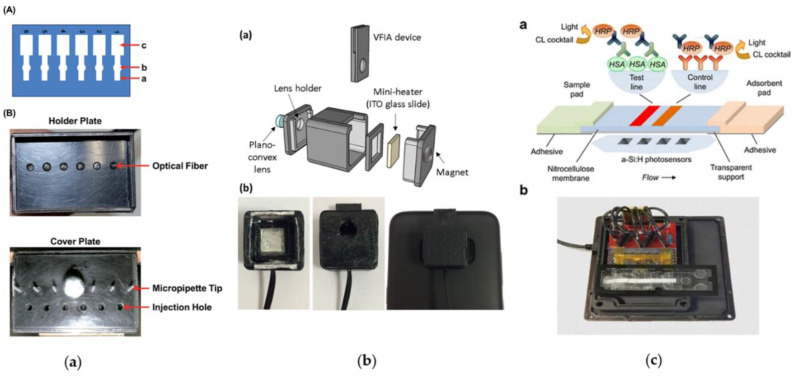
Light detection technologies integrated with CL paper-based immunoassay devices: (**a**) a miniaturized chemiluminescence detection system for a microfluidic paper-based analytical device and its application to the determination of chromium (III) (A): (a) injection zone, (b) reaction zone, and (c) waste zone. (B) holder: upper, holder of the μPAD. The optical fibers are located at the positions of the reaction zone. Lower, the cover of the holder. The CL reagents were introduced from the injection holes located at the μPAD injection zones. Reproduced from Ref. [79] with permission from The Royal Society of Chemistry. (**b**) Scheme of the integrated thermochemiluminescent vertical flow immunoassay device: (a) Scheme of the integrated TCL-VFIA device; (b) inside view of the mini-dark box (left), mini-dark box with VFIA device inserted (center), configuration for TCL signal acquisition (right). Reproduced with permissione from Ref [84]. Copyright 2018 Elsevier; (**c**) scheme of the CL-LFIA strip showing the assay principle, the position of the photosensors below the strip, and a picture of the complete device, comprising the PDMS microfluidic cartridge with integrated a-Si:H photosensors and the custom readout electronics: (a) Scheme of the CL-LFIA strip showing the assay principle (top) and the position of the photosensors below the strip (bottom). (b) Picture of the complete device, comprising the PDMS microfluidic cartridge with integrated a-Si:H photosensors and the custom readout electronics. Reproduced with permission from Ref [95]. Copyright 2016, Springer-Verlag Berlin Heidelberg.

A conventional detector for CL measurements is the photomultiplier tube (PMT), which provides the highest sensitivity and, due to its size and cost, is typically used as benchtop laboratory equipment. However, recent technological advances allowed the use of portable and compact PMT-based detectors, as reported by Alahmad et al. [79]. They developed a miniaturized detection system for CL reaction, generated on μPAD using optical fibers. Wax printing technology was employed for developing the μPAD which comprised six separate parallel channels. Each channel was composed of three different zones (injection, reaction and waste, respectively). The CL signal was acquired by placing the μPAD on a plastic holder equipped with six optical fibers connected to a small PMT module (Figure 5a).

Recently, several detectors combining adequate sensitivity with portability have been proposed in order to overcome the main limitation of the relatively weak CL signal and to allow the development of ultrasensitive POCT assays [33]. For example, CL-based biosensors were implemented with a new generation of (thermally cooled) back-illuminated (BI) CCD integrated with LFIA technologies [73,75,76,80]. In these examples, the CL measures were conducted in the “contact imaging” approach which consists in placing the LFIA strip directly in contact with CCD sensors through a fiber optic faceplate in order to maximize the photon collection efficiency [81].

As a low cost, easy-to-use and compact alternative, the smartphone complementary metal-oxide semiconductor (CMOS) camera has been reported as a CL detector for different POC applications. Indeed, smartphone-based biosensors have great potential since their connectivity and data processing capabilities can be exploited to perform on site and POC analyses [78,82]. Several examples have been reported in literature coupling CL-LFIA with this innovative sensing platforms (Figure 5b) [83,84]. All the mentioned biosensors showed performances that are comparable to those obtained with reference methods. A digital camera was used by Xue et al. [85] for the development of a portable and low-cost diagnostic biosensor based on BL detection. In particular, the authors proposed a method that can be adapted to different applications for transforming antibodies into ratiometric, bioluminescent sensor proteins for the quantitative detection of target analytes. The approach is based on the genetic fusion of antibody fragments to NanoLuc luciferase and SNAP-tag. SNAP-tag was labeled with a synthetic fluorescent competitor of the antigen. According to the competitive immunoassay method, when the antigen binds to the antibody, the displacement of the tethered fluorescent competitor occurs. This phenomenon brings about the disruption of the bioluminescent resonance energy transfer (BRET) between the luciferase and fluorophore. The semisynthetic sensor offers the possibility to tune the response range (submicromolar to submillimolar) and to obtain a large dynamic range. Moreover, it allows to quantify analytes simply by spotting the samples on a paper support and exploiting a digital camera for signal acquisition.

Generally, a robust BRET sensor is characterized by the ability to control ligand-induced switching between a high BRET-state and a low BRET-state, in which luciferase and the FL acceptor are in close proximity or are well separated, respectively. Ni et al. introduced a new class of ratiometric BL sensor proteins based on the competitive intramolecular complementation of split NanoLuc luciferase as an alternative to the classical BRET sensor design. As proof of concept the authors developed a blue-red light emitting sensor protein for the detection of anti-HIV-p17 antibodies. In particular, the sensor format (NB-LUMABS) is designed with a single copy of the large fragment (LB) fused to two copies of the small fragment (SB) yielding a protein switch that can exist in two conformations, where either the N- or the C-terminal SB binds to the LB and reconstitutes luciferase activity. The red-emitting fluorophore, coupled to one of the SBs, allows an efficient BRET with a consequently emission of red light in only one of the two conformations, while the intramolecular interaction of the fluorescently labeled SB is disrupted by the bivalent binding of the antibody. This allows for the reconstitution of NanoLuc by the nonfluorescently labeled SB, resulting in a color transition from red to blue [86].

As an alternative, the integration of relatively inexpensive thin-film photosensors in the analytical device was also investigated since this approach could reduce costs, electrical power consumption and memory storage space. Among the different reported technologies, amorphous silicon thin-film photodiodes [87,88], organic photodiodes [89,90], carbon nanotubes coated with photovoltaic polymers [91] and metal–semiconductor–metal photodetectors [92] have gained great interest. By optimizing chip design, sensor architecture and readout electronics, photon collection efficiency can be increased to achieve analytical performances comparable with CCDs [93,94].

Recently we developed a disposable cartridge for CL-LFIA with integrated amorphous silicon (a-Si:H) photosensors array for detecting human serum albumin (HSA) in urine samples [95]. The proposed approach is based on an indirect competitive immunoassay in which HRP acts as a tracer that is detected upon the addition of the luminol/enhancer/hydrogen peroxide CL cocktail. A PDMS cartridge that housed the LFIA strip and the reagents necessary for the CL immunoassay was optically coupled to the array of a-Si:H photosensors which were deposited on a glass substrate. This configuration ensures to obtain an integrated analytical device controlled by a portable read-out electronics.

The analytical performances of this biosensor demonstrate that implementing the CL-LFIA technique with the a-Si:H photosensors array allows to obtain a compact, sensitive and low-cost system for CL-based bioassays with a wide range of applications for in-field and point-of-care bioanalyses. Furthermore, multiplexed bioassays can be easily developed by exploiting arrayed photosensors with custom geometries (Figure 5c).

## 6. Conclusions

The combination of immunoassays with CL detection and paper-based technology represents an ideal solution for the realization of last generation analytical devices for POC applications. Indeed, it is possible to obtain high selectivity and specificity even in complex and untreated matrices typical of the biospecific molecular recognition methods, together with the simplicity of the instrumentation necessary to measure the light signal. Furthermore, paper-based devices add great versatility, ease of use and compactness of the entire system. Even though significant progresses are continuously appearing in literature, some limitations are still hindering their spread in the market. Due to the limited stability over time of the bioreagents and the need to add reagents manually while performing the analytical protocol, the number of commercial paper-based CL-immunoassays remains low, having difficulties meeting the end-users’ needs. This issue is even more relevant for BL immunosensors, explaining why very few examples have been reported in the literature. The approach of Hall et al., who developed a stable lyophilized cake integrating all reagents and substrates required for the BL reaction, surely represents one of the most promising strategies that could solve the issues related to scarce shelf-life of BL sensors and boost their implementation in the market [30].

Researchers are now focusing their efforts on improving these systems for fulfilling the ideal POC biosensors requirement for the transition of this technology from laboratory to the market. Innovative materials and solutions are proposed in order to make these devices manageable by end-users, enabling the development of several analytical devices for a wide variety of applications.

The expected diffusion of these bioanalytic tools in commerce and everyday life will pave the way for a great change in the field of analytical chemistry, making POC devices more accessible to everyone, significantly reducing time and costs for analysis, thus allowing the widespread availability of these kind of tools.

## Figures and Tables

**Table 1:** Nanomaterials and related mechanism proposed for CL enhancement.

Nanomaterial	Enhancement Mechanism Exploiting Luminol/H_2_O_2_ CL System	Features	Ref.
Metal nanoparticles (AgNPs, AuNPs, PtNPs, etc.)	MNPs catalyze the decomposition of H_2_O_2_ leading to the formation of a hydroxyl radical, which reacts with a luminol anion and HO_2_^−^. These species are involved in the production of a luminol radical and a superoxide anion leading to light emission. The radical generation and electron transfer processes take place on the surface of the MNPs, which are therefore responsible for the facilitation of these processes.	chemical reactivity catalytic properties surface properties biocompatibility ease of self-assembly	[50]
Quantum dots	Quantum dots act first by decomposing H_2_O_2_ to generate free radicals and then promoting CL by energy transfer and electron transfer annihilation effects.	catalytic properties for redox reactions size-dependent catalytic action controllable charge-electron-transfer events, biocompatibility	[41]
Carbon nanomaterials(carbon nanoparticles (CNPs), graphene, graphene oxide (GO) and carbon nanotubes (CNTs))	A possible mechanism involves the reaction between carbon materials with π-rich electronic structures and luminol allowing the formation of the activated transition complex. This complex may accelerate electron-transfer processes during the luminol-dissolved oxygen CL reaction.	low toxicity, environmental friendliness low cost simple synthetic routes tunable catalytic activities depending on surface functionalization	[51]

**Table 2:** Technologies for CL detectors implemented into paper-based portable immunosensors.

Detector for CL Paper-Based Immunosensor	Detection Principle	Pros	Cons
Photomultiplier tube (PMT)	Each PMT can detect just one wavelength. It captures emitted photons and its photocathode layer, then converts these photons to electricity. Dynodes are then used to multiply this charge multi-fold, making it readable for the instrument.	Excellent signal amplification Short response times High sensitivity Low noise	Expensive Large size Each wavelength needs a specific element High energy consumption Low design flexibility
Charged coupled device (CCD)	In a CCD image sensor, pixels represent the basic building blocks, and they are composed of p-doped metal–oxide-semiconductor (MOS) capacitors. Pixels allow the conversion of incoming photons into electron charges at the semiconductor-oxide interface; the CCD is then used to read out these charges.	Good sensitivity Small dark current Wide range of wavelength Compact High image quality	High power consumption Difficult system integration More expensive than CMOS
Complementary metal-oxide semiconductor (CMOS) sensor	CMOS has emerged as alternative to CCD. Differently from CCD, each pixel sensor unit has a photodetector.	Low-cost Wide range of wavelength Easy implementation into portable device (e.g., smartphone’s camera) Low power consumption High speed	Lower sensitivity respect CCD Large dark current
Thin-film photosensors	There is a wide variety of photosensors based on different materials but they are typically based on a p–n junction that converts light photons into current. The absorbed photons make electron–hole pairs in the depletion region.	Low-cost Easy implementation into portable device Low power consumption Possibility to develop disposable platforms based on thin-film photosensors Custom geometries can be developed	Large dark current Low thermal stability Low sensitivity
